# Comparison of Asbestos Victim Relief Available Outside of Conventional Occupational Compensation Schemes

**DOI:** 10.3390/ijerph18105236

**Published:** 2021-05-14

**Authors:** Kwang Min Lee, Lode Godderis, Sugio Furuya, Yoon Ji Kim, Dongmug Kang

**Affiliations:** 1 Department of Occupational and Environmental Medicine, Pusan National University Yangsan Hospital, Yangsan 50612, Korea; lightstone@pusan.ac.kr; 2 Centre for Environment and Health, University of Leuven, 3000 Leuven, Belgium; lode.godderis@kuleuven.be; 3 IDEWE, Knowledge, Information and Research Center, 3001 Heverlee, Belgium; 4 Japan Occupational Safety and Health Resource Center, Tokyo 136-0071, Japan; joshrc@joshrc.net; 5 Department of Preventive, and Occupational & Environmental Medicine, Medical College, Pusan National University, Yangsan 50612, Korea; harrypotter79@pusan.ac.kr; 6 Environmental Health Center of Asbestos, Pusan National University Yangsan Hospital, Yangsan 50612, Korea

**Keywords:** asbestos, asbestos related disease, compensation, relief, comparison, system, non-occupational exposure, occupational exposure, claim, compensation amount

## Abstract

The asbestos victim relief schemes were introduced to resolve the issue of victims of asbestos-related diseases not receiving compensation through conventional legal orders. This article seeks to derive the differences and commonalities of various asbestos victim relief schemes available outside of the conventional occupational compensation system along with a systematic understanding and to propose plans for improvement through a comparative study. After the degree of asbestos exposure, the population, and the period of implementation were corrected, the recognized claims of the total of conventional occupational compensation schemes and the asbestos victim relief schemes could be ranked in the order of South Korea (KOR) (1867, total), France (FRA) (1571), Japan (JPN) (966), KOR (847, asbestosis grade 2,3 excluded), the United Kingdom (GBR) (670), and the Netherlands (NLD) (95). The average amount of compensation per person, in the case of mesothelioma, was higher in the order of FRA (4.60 times), KOR (1.46 times), GBR (1.03 times), and NLD (0.73 times) of the median income per year. The differences between countries were largely caused by the purpose of institutional design and influenced by the level of qualification, the existence of an expiration date, type of disease, type of benefit, level of judgment criteria, the existence of a procedure for appeals, and recognition rate (GBR: 102%, FRA: 84%, NLD: 81%, JPN: 76%, KOR: 73%, and BEL: 54%). Based on this analysis, suggestions could be made regarding the expansion of disease types, benefit types, and the overall review of judgment criteria.

## 1. Introduction

According to the World Health Organization (WHO), approximately 125 million people worldwide are exposed to asbestos at work, and 1.52 million disability adjusted life years (DALYs) are reported to be caused by asbestos [1]. The estimated number of fatalities due to occupational asbestos-related diseases (ARD) is 232,562, and non-occupational ARD is 22,064 [2].

Many countries have not intervened and left individuals to resolve the ever-increasing social disputes caused by ARD through civil proceedings [3,4]. However, this solution has had several problems [5,6]: (i) it is difficult to identify the perpetrator; (ii) in many cases, the perpetrator does not have the ability to compensate or does not longer exist; (iii) the social cost is high; (iv) it takes a long time to receive compensation. As social conflicts were not resolved and in fact, increased due to these problems, countries having worker’s compensation systems used it, as such, to compensate victims of occupational ARD [7]. In the Netherlands (NLD), where there is no worker’s compensation system, Compensation for Asbestos Victim (TAS) was established in 2000 for employees and members of their household. Nevertheless, many occupational victims were not compensated due to the insufficient coverage of conventional occupational compensation schemes and the difficulty in proving occupational damage given the characteristics of ARD. The non-occupational victims were still outside the compensation system [8,9,10]. Based on the awareness of this problem, France (FRA) implemented the Fund for the Compensation of Asbestos Victims (FIVA) as an asbestos victim relief scheme in 2002 based on a court judgment that the state is responsible for asbestos-related health issues and the concept of social solidarity by virtue of which the benefits of the use of asbestos were shared by society. Since then, several countries have implemented similar systems [11]. The countries implementing asbestos victim relief schemes worldwide are FIVA of FRA (2002), Asbestos Health Damage Relief System of Japan (JPN) (2006), Asbestos Fund (AFA) of Belgium (BEL) (2007), Non-work-related Compensation for Asbestos Victims (TNS) of NLD (2007), Diffuse Mesothelioma Payments (the ‘2008’ scheme) of the United Kingdom (GBR) (2008), and Asbestos Injury Relief System of South Korea (KOR) (2011).

There are published articles and studies of the asbestos victim relief scheme of each country [11,12]. However, the number of articles and studies on related topics is small because the period of implementation is short, and only six countries are implementing these systems. Moreover, the currently reported literature simply introduces the background, history, and status of each country’s system. Comparative studies, on the other hand, allow us to identify the universality and specificity of these systems and to identify the causes that make the universality and specificity [13]. This is meaningful in that it allows us to clarify the goals that the government should achieve to solve public problems and the guidelines for action plans. However, to ensure the credibility of the comparative study, the premise must take into account differences in various factors such as population, degree of exposure to asbestos, the implementation period of the system, income level, and price. Additionally, related systems within the overall framework of the social security system, such as the occupational ARD compensation system, medical payment system, etc., should be considered. Unfortunately, it is hard to find articles that have compared and comprehensively analyzed the various systems taking these factors into account.

The purpose of this study is to collect and provide the latest information of each country implementing an asbestos victim relief scheme and to derive characteristic commonalities and differences by comparing and analyzing the various asbestos victim relief schemes considering various factors in totality. Based on the available data, principles and discussions that should be considered in the asbestos victim relief schemes along with recommendations have been provided. 

## 2. Materials and Methods

The asbestos victim relief schemes for each country are as follows: FIVA (FRA), Asbestos Health Damage Relief System (JPN), AFA (BEL), TNS (NLD), the ‘2008’ scheme (GBR), Asbestos Injury Relief System (KOR). The analysis covers relevant laws, data, research, and studies published for FRA, JPN, BEL, NLD, GBR, and KOR to compare and analyze asbestos victim relief schemes. If more data were needed due to the limitations of publicly available data, as in the case of GBR’s the ‘2008’ scheme and KOR’s Industrial Accident Compensation Insurance, the necessary information could be obtained through a request for information disclosure to the relevant organization under the Freedom of Information Act. Specific topics covered are as follows: system, qualification, expiration date, disease, type of benefit, judgment criteria, appeals, recognition rate, number of recognized claims, and compensation amount. 

### 2.1. Recognition Rate

The recognition rate was obtained by $\frac{\mathrm{the}\;\mathrm{number}\;\mathrm{of}\;\mathrm{recognized}\;\mathrm{claims}}{\mathrm{the}\;\mathrm{number}\;\mathrm{of}\;\mathrm{applications}}$ during the entire period of operation of the system. The rates of individual countries are analyzed as follows: FRA: Only the recognition rate of all cases (new cases + deterioration of existing recognized disease) is taken since data is not separately available for new cases. This means that what counts as a single case in other countries may correspond to multiple case counts in FRA. 2002–2018, total $\frac{237,530}{282,157}$ [14]. JPN: 2006–2018, mesothelioma $\frac{11,860}{13,983}$, lung cancer $\frac{1911}{3661}$, asbestosis $\frac{78}{433}$, diffuse pleural thickening $\frac{163}{399}$ [15]. BEL: 2007.04–2018, mesothelioma $\frac{2287}{2456}$, asbestosis $\frac{309}{2402}$, diffuse pleural thickening $\frac{513}{858}$. NLD: The recognition rate for the total of TAS and TNS is known since the number of applications is disclosed regardless of the type of scheme. 2007.12–2019, mesothelioma $\frac{5461}{6397}$, asbestosis $\frac{218}{576}$ [16,17]. GBR: Data on the number of applications for GBR was only disclosed for 2017–2019. GBR provides data rounded to the nearest ten. 2017–2019, mesothelioma $\frac{1280}{1260}$. KOR: Out of the total number of applications, 118 (mesothelioma), 87 (lung cancer), 130 (asbestosis), 6 (diffuse pleural thickening) cases were excluded since those were subject to other laws. 2011–2019, mesothelioma $\frac{1074}{1256}$, lung cancer $\frac{623}{1042}$, asbestosis $\frac{2436}{3316}$, diffuse pleural thickening $\frac{4}{35}$ [18].

### 2.2. Number of Recognized Claims

As the cause of mesothelioma is mostly asbestos exposure, it roughly reflects the degree of asbestos exposure in a country. Therefore, the population and degree of asbestos exposure were corrected by the crude mesothelioma mortality rates per 100,000 people of males, and all ages by country (FRA: 2.3 (2015), JPN: 2.1 (2016), BEL: 3.7 (2016), NLD: 5.4 (2016), GBR: 6.4 (2016), KOR: 0.3 (2016)) [19]. Since there is a difference in the implementation period of the asbestos victim relief scheme in each country and each disease, this was corrected in units of one year. FRA: The number of recognized claims based on all cases (new cases + deterioration of existing accreditations) is only disclosed. Therefore, the number of newly recognized cases was estimated by multiplying the number of newly applied cases by the recognition rate of all cases. 16 years (2003–2018, all diseases), JPN: 13 years (2006–2018, mesothelioma, lung cancer), 8.5 years (2010.07–2018, asbestosis, diffuse pleural thickening), BEL: 11.75 years (2007.04–2018, all diseases), NLD: The number of recognized claims of 2016 and 2017 are not known. Therefore, it was calculated excluding these years. 10.08 years (2007.12–2015, 2018–2019, mesothelioma), 3.75 years (2014.04–2015, 2018–2019, asbestosis), GBR: 11.25 years (2008.10–2019, all diseases), KOR: 9 years (2011–2019, mesothelioma, lung cancer, asbestosis), 6 years (2014–2019, diffuse pleural thickening). The correction was made by $\frac{\mathrm{the}\;\mathrm{acutal}\;\mathrm{number}\;\mathrm{of}\;\mathrm{recognized}\;\mathrm{claims}}{\mathrm{the}\;\mathrm{rate}\;\mathrm{of}\;\mathrm{incidence}\;\mathrm{of}\;\mathrm{mesothelioma}\;\mathrm{per}\;100,000\;\mathrm{people}\;\times \;\mathrm{the}\;\mathrm{implementation}\;\mathrm{period}\;\left(\mathrm{year}\;\mathrm{unit}\right)}$. Only KOR has an asbestos victim relief scheme that grades asbestosis (grades 1,2,3) and provides a differential compensation amount. The number of recognized claims may be overestimated when compared to other countries since 1st-grade asbestosis in KOR is equivalent to the recognized asbestosis of other countries in terms of the level required for recognition. Therefore, the number of recognized asbestosis claims were calculated (i) excluding asbestosis grades 2 and 3, (ii) the total number of recognized claims of asbestosis, respectively.

Statistics of the conventional occupational compensation scheme include data from the following: Industrial Accident Compensation Insurance (JPN), TAS (NLD), Industrial Injuries Disablement Benefit (GBR), and Industrial Accident Compensation Insurance (KOR). Other minor systems compensating occupational ARD are not considered.

### 2.3. Compensation Amount

The average compensation amount per person by country was compared. The average compensation amount per person for each disease was calculated by $\frac{\mathrm{total}\;\mathrm{compensation}\;\mathrm{amount}\;}{\mathrm{the}\;\mathrm{number}\;\mathrm{of}\;\mathrm{recognized}\;\mathrm{claims}}$ during the entire period. FRA: Since the total compensation amount for each disease is disclosed, published data were used [14]. JPN: As the total compensation amount is not disclosed by each disease but disclosed as a whole, the average amount of compensation per person was not known for each disease, but only as a whole for all diseases. 2006–2018, $\frac{46,326,804,000\;¥}{14,012}$ [15]. NLD: The number of recognized claims of 2016 and 2017 is not known. Therefore, it was calculated excluding these years. 2007.12–2015, 2018–2019, mesothelioma $\frac{23,101,289\;€}{1213}$, asbestosis $\frac{20,355\;€}{1}$ [16,17]. GBR: GBR provides data rounded to the nearest ten. 2008.10–2019, $\frac{100,756,478₤}{5220}$ [20]. KOR: 2011–2019, mesothelioma $\frac{41,483,000,000\;₩}{1074}$, lung cancer $\frac{23,890,000,000\;₩}{623}$, asbestosis grade1 $\frac{8,201,000,000\;₩}{243}$, asbestosis grade2 $\frac{10,031,000,000\;₩}{799}$, asbestosis grade3 $\frac{7,482,000,000\;₩}{1394}$, diffuse pleural thickening $\frac{112,000,000\;₩}{4}$ [18]. Then to correct the income level, it was divided by the median income (FRA: 22,610 € (2017), JPN: 2,443,000 ¥ (2015), GBR: 18,713 ₤ (2017), NLD: 26,200 € (2016), KOR: 26,430,000 ₩ (2017)) for each country [21].

## 3. Results

### 3.1. System

In countries where worker’s compensation exists as a part of the social security system, occupational ARD is compensated within this system and a new system was created to compensate asbestos victims outside the conventional occupational compensation schemes. These countries are FRA, JPN, BEL, GBR, and KOR. Specifically, in FRA, a worker’s compensation system exists but all ARD are equally compensated regardless of whether the exposure is occupational or non-occupational through a new system called FIVA. In other words, in the case of occupational ARD, after receiving compensation from the worker’s compensation system, additional compensation can be received through FIVA. Unusually, BEL reviews applications for asbestos victim relief through worker’s compensation schemes. In the UK, the worker’s compensation system handles occupational ARD and the ‘2008’ scheme handles asbestos victims outside the worker’s compensation system. However, Diffuse Mesothelioma Payment Scheme (DMPS) exists as an occupational ARD compensation system for cases where it is unable to trace the employers or the insurers responsible for a victim due to various limitations even though it is an occupational ARD. The difference between the ‘2008’ scheme and the DMPS is that DMPS has a higher compensation amount, but it is more difficult to be recognized as a victim under this scheme. NLD does not have a compensation system related to occupational diseases for workers. For this reason, it was decided to establish a coordination body through a grand compromise between workers, employers, the government, asbestos victims, and insurance companies. The coordination body called Institute for Asbestos Victims (IAS) operates TAS for employees and members of their household in 2000. Afterwards, IAS operates TNS for asbestos victims by outside of TAS. First, if the application to the IAS is recognized, compensation will be paid at a flat rate regardless of whether it is TAS or TNS. Thereafter, in the case of TAS, if the IAS can identify the employer responsible for the occupational exposure, it will investigate whether the employer is liable to the victim. If the employer is liable, IAS acts as an intermediary to facilitate payment of additional compensation by employers. However, IAS merely makes a recommendation that is not legally binding, as can be seen from the term used coordination body [22] (Table 1).

Concerning litigation rights, victims in JPN, NLD, GBR, and KOR do not lose the right to litigation after they have benefited from the asbestos victim relief scheme. However, in FRA and BEL victims are not permitted to file civil suits on the same matter if they have received compensation from asbestos victim relief (Table 1).

In FRA, BEL, and NLD, the overall framework for budget management is that the funds of the social security system are the main source and government subsidies are used as secondary sources of funding. Specifically, FRA operates with subsidies mainly from the worker’s compensation system, and government subsidies are used as secondary resources. BEL operates with subsidies from the worker’s compensation system, the government, the National Institute for the Social Security of the Self-employed (NISSE), and various donations. In NLD, the social insurance bank (SVB), which is in charge of the operation of national social insurance, is responsible for the budget. Unlike the countries mentioned above, JPN and KOR have established new funds primarily collected from the members of the workers’ compensation system and a few asbestos-related businesses (4 companies each in JPN and KOR), and government subsidies are used as secondary resources. In GBR, the ‘2008’ scheme is being financed from amounts recovered from later awards of civil compensation made to people who have already received a payment under either the scheme or the Pneumoconiosis etc. (Workers’ Compensation) Act 1979 (Table 1). 

### 3.2. Qualification

In FRA, victims must apply within 10 years from the date of the first medical certificate, which is issued at the time of diagnosis of the disease known to be caused by asbestos exposure. When applying for an exacerbation of an existing recognized disease, the application must be made within 10 years from the date of the first medical certificate related to the exacerbation. In JPN, BEL, and KOR, there is no time limit for applying when a victim is alive, but in the case of a deceased person who had not applied when alive, the time limit is set at 15 years (JPN, KOR), half a year (BEL), from the date of death. In NLD, victims can only apply if they are alive and should have lived at least 10 consecutive years in NLD, 10 to 60 years prior to the application. In the GBR, victims must apply within one year of the date of diagnosis of ARD or one year from the date of death (Table 1).

If a person recognized by the asbestos victim relief scheme has another ARD, NLD makes it impossible to reapply for further compensation. GBR also recognizes only one disease (mesothelioma), which makes re-application essentially impossible. Victims in other countries can receive further compensation through re-application (Table 1). 

If the victim has received compensation from the occupational ARD compensation system, in JPN, NLD, GBR, KOR, he or she is not eligible for asbestos victim relief. In BEL, even in such cases, it is possible to apply for asbestos victim relief. In FRA, it is possible to apply to FIVA even if the victim has received compensation from the worker’s compensation system. However, if he or she has already received compensation from FIVA due to occupational ARD, it is not possible to re-apply to FIVA for the same reason. In the case of duplicate compensation, the compensation amount received from the related system for the corresponding disease is deducted (Table 1).

### 3.3. Expiration Date

In FRA and BEL, there is no limitation on the validity period. JPN and KOR set the expiry period as five years. After that, the victim can apply for renewal if further medical treatment is necessary. The validity will be extended if approved. In GBR and NLD, there is structurally no expiration date as the form of compensation is a lump sum (Table 1).

### 3.4. Disease

There are open, closed, and mixed types of systems for managing recognized diseases that differ from country to country. Open type refers to the evaluation of individual applicants without limiting the types of recognized diseases. The closed type creates a list of diseases and limits recognized diseases to those specified in the list. The mixed type operates the open type and the closed type systems together.

Currently, no country is using the open type. FRA is the only country operating a mixed type system that opens the possibility of compensation for all diseases without imposing a limitation on the disease type. Other countries use the closed type and recognize several diseases in the decreasing order of BEL, JPN = KOR, NLD, and GBR (Table 1).

### 3.5. Type of Benefits

FRA aims for complete compensation, so its system is characterized by many benefits to compensate for various types of damage. Additionally, individual compensation is provided according to the specific situation without using a flat rate. All the compensation is based on the documents provided by the victim. Economic damages such as loss of income, medical expenses, other expenses are paid to the extent incurred; non-economic damages are calculated individually by FIVA’s lawyers. In BEL, if recognized when alive, mesothelioma is compensated in the form of reimbursement of medical expenses, a lump sum, subsistence allowance (flat-rate) and attendance allowance, and compensation for any other disease is in the form of medical expenses reimbursement, subsistence allowance (in proportion to % of the disability) and attendance allowance. Since NLD and GBR pay only a lump sum, there is only one type of benefit. JPN and KOR aim to assist victims only during the treatment period, which achieves its purpose in the form of setting a specified expiration date. Therefore, no benefits can be received if the specified expiration date cannot be extended by renewal (Table 2).

### 3.6. Judgement Criteria

When the victim applies to the asbestos victim relief scheme, the evaluation to determine whether the victim can be recognized is divided into medical criteria and exposure criteria on the premise that the applicant’s disease has been diagnosed. The medical criteria consist of the severity of symptoms and the medical findings that indicate that the disease was caused by asbestos. The exposure criteria refer to an epidemiological investigation into asbestos exposure and the amount of exposure.

FRA, JPN, BEL, and NLD use both medical and exposure criteria. In FRA, the relevant details were not disclosed. In JPN, both medical criteria and exposure criteria should be satisfied for asbestos and diffuse pleural thickening, but it is enough if only medical criteria are met for lung cancer. BEL uses medical criteria as a complementary measure in cases where it is difficult to make a judgment based on exposure criteria alone or where it is impossible to investigate the exposure because of the lack of relevant information. NLD requires that both medical and exposure criteria be satisfied. KOR makes a judgment only on medical criteria. In GBR, neither medical nor exposure criteria exist.

#### 3.6.1. Medical Criteria

A common characteristic is that there are no specific medical criteria to implicate asbestos as the cause of mesothelioma as the condition itself is clearly indicative of asbestos exposure [23]. In JPN and KOR, medical criteria must be very specific. In KOR, the level of compensation varies according to the severity of the disease (grades 3, 2, and 1), progressively lowering the criteria for asbestosis recognition, in more severe cases. The medical criteria for asbestosis in KOR indicated in Table 3 are referred to as the 3rd grade, which has the least stringent standards and compensation; the specific standards for grades 3, 2, and 1 are separately presented in Table 4 [24]. Contrary to JPN and KOR, where medical criteria are very specific, BEL’s medical criteria are not specific because they are used only as an auxiliary means of measuring exposure (Table 3).

#### 3.6.2. Exposure Criteria

In JPN, acceptance of asbestosis is based on the following: (i) Confirmation that there is sufficient evidence of asbestos exposure through a quantitative evaluation based on the applicant’s job history. This history is assessed through interviews and a perusal of various documents detailing the name and location of the workplace, work content, work hours, work period, etc. If the applicant’s job history is not clear, this exposure criteria can be replaced by medical criteria 3 of lung cancer. Diffuse pleural thickening should satisfy two criteria, (i) mentioned above, and (ii) at least three years duration of work related to asbestos exposure.

BEL (i) checks whether there is sufficient evidence that the asbestos exposure has occurred in BEL based on information about the applicant’s occupational history. There are no other criteria for mesothelioma. However, for the other diseases (lung cancer, asbestosis, diffuse pleural thickening, laryngeal cancer), additional exposure criteria must be satisfied. Lung cancer must satisfy (i) above and (ii) asbestos cumulative exposure exceeding 25 fiber-years/cc. This is based on the Helsinki criteria accepted by international consensus to determine whether or not lung cancer is caused by asbestos. For ease of calculation, it is assumed that workers who have been engaged in a specific industry or job for more than 10 years prior to 1985 satisfy the criteria. For other industries or jobs, it is calculated as the average concentration of asbestos fibers in the air at the workplace multiplied by the actual duration of exposure, and then aggregated taking into account each exposure. The average concentration of asbestos fiber in the air at the workplace is determined based on results measured at a workplace similar to the applicant’s occupational history. The actual exposure period is calculated by considering only the work hours that include asbestos exposure. If the actual working hours cannot be estimated in detail, it is calculated as 8 h/day, 5 days/week, 20 days/month or 4 weeks/month, 12 months/year. (iii) The latent period must be 10 years or longer. Asbestosis, diffuse pleural thickening, and laryngeal cancer cases should satisfy criteria (i), (iii) above, and (iv) cumulative exposure exceeding 25 fiber-years/cc by a qualitative evaluation of asbestos exposure instead of (ii). This is because there is no internationally accepted quantitative standard for determining whether each disease is caused by asbestos [25]. 

In the NLD, there are no exposure criteria for mesothelioma. However, for asbestosis, (i) the latent period must be 15 years or longer. Besides, (ii) asbestos cumulative exposure must exceed 5 fiber-years/cc. This is based on a protocol created by the Health Council of the Netherlands in 1999 [26]. If the victim’s job cannot be linked to the proposed protocol, a quantitative evaluation is performed based on the information in the applicant’s job history, using data from NLD and overseas. The applicant’s job performance period is calculated in units of 20 days or 4 weeks = 1 month based on the actual working period.

### 3.7. Appeals

In JPN, KOR, and NLD, processes for appeals other than litigation exist for the contest of disapproval by the relevant institution’s operating system, so it is designed to allow re-examination if the applicant so wishes. JPN has a second review system, while KOR and NLD have a third review system. Conversely, in FRA, BEL, and GBR, there is no procedure for the victim to file an objection to the institution’s disapproval due to the absence of the right to appeal other than litigation.

### 3.8. Recognition Rate

**Figure 1:**
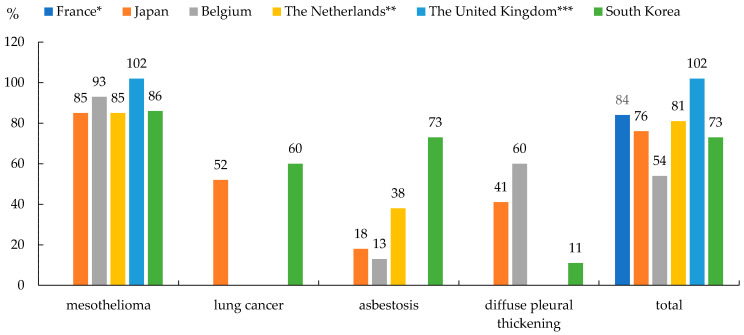
Recognition rate of mesothelioma, lung cancer, asbestos, pleural thickening, total ARD cases during the entire period of operation of the system. * Recognition rate of all cases (new cases + deterioration of existing recognized disease). ** Recognition rate for the total of Compensation for Asbestos Victims (TAS) and non-work-related Compensation for Asbestos Victims (TNS) from 2007.12 to 2019. *** 2017–2019.

The recognition rate of the total was in the descending order of GBR, FRA, NLD, JPN, KOR, and BEL. The mesothelioma recognition rate was in the descending order of GBR, BEL, KOR, and JPN = NLD. Similarly, lung cancer was in the order of KOR and JPN, asbestosis was in the order of KOR, NLD, JPN, and BEL and pleural thickening was in the order of BEL, JPN, and KOR (Figure 1). 

### 3.9. Number of Recognized Claims

Based on the overall disease count, the recognized claims of the total of conventional occupational compensation schemes and the asbestos victim relief schemes during 2016–2018 were in the following order: KOR (total), FRA, JPN, KOR (excluding asbestosis grades 2 and 3), GBR, and NLD. It was not possible to analyze the number of recognized cases by disease in FRA because it was not disclosed. Mesothelioma was recognized in the descending order of JPN, GBR, KOR, and NLD. Lung cancer was in the order of KOR, JPN, and GBR. Asbestosis was in the order of KOR (total), KOR (excluding asbestosis grades 2 and 3), GBR, JPN, and NLD. Diffuse pleural thickening was in the order of GBR, JPN, and KOR (Table 5).

The ratio of the number of recognized claims of the conventional occupational compensation scheme to that of the asbestos victim relief scheme by country showed that the ratio was high and in the descending order of GBR, NLD, JPN, KOR (excluding asbestosis grades 2 and 3), and KOR (total) (Table 6).

### 3.10. Compensation Amount

The average compensation amount per person by country was compared. In the case of mesothelioma, the ratio of the average compensation amount per person to the median income per year was 4.60 in FRA, 1.46 in KOR, 1.03 in GBR, and 0.73 in NLD. The corresponding ratio for lung cancer in FRA was 4.92 and KOR was 1.45. For asbestosis, the ratio for FRA was 1.75, KOR (grade 1) was 1.28, NLD was 0.78, KOR (grade 2) was 0.48, and KOR (grade 3) was 0.20. In Japan, the average compensation amount per person based on the total number of recognized victims was 1.35 times the median income, but it was not subdivided by disease classification. In BEL, the average compensation amount per person was not known due to the lack of data disclosure (Table 7).

## 4. Discussion

### 4.1. System

In the case of occupational exposure, the responsibility can be assigned to the perpetrator since this entity can be specified. This is the reason why most countries operate their occupational ARD compensation system and the asbestos victim relief scheme differently. FRA, on the other hand, compensates occupational ARD through the worker’s compensation system, but it is also possible to apply to FIVA for more compensation. In other words, FRA does not classify victims according to the route of exposure and gives the same for treatment for any compensation claim. FRA recognizes that only the route of exposure is different while the health damage caused by exposure is the same, and the benefits are shared not only by employers but also by the society at large [8].

Concerning lawsuits, the difference between FRA, BEL (where the victim is required to renounce any right to further claims to obtain compensation for the same damages), and other countries can be interpreted as originating from the difference between complete compensation (FRA, BEL) and burden reduction (the other countries) [8].

FRA, BEL, NLD, JPN, and KOR are similar in terms of the entity in charge of the fund burden and they differ only in methods (FRA, BEL, and NLD where the existing social security funds are used as compared to JPN and KOR where new funds were created). The difference is that JPN and KOR put an additional burden on asbestos-related businesses. In contrast, GBR does not use funds from the existing social security system and has not established a new fund either. GBR operates only with redemption due to the recovery of benefits. One of the most important factors for the stable operation of the system is fund stability. The fact that the number of recipients of the asbestos victim relief schemes is increasing as ARD increases cause systemic changes according to various demands and this can affect the stability of the fund. The use of funds from the existing social security system or securing the budget through the establishment of new funds is predictable and continuous. Those methods are better than the unpredictable and temporary fund management methods of GBR in terms of fund stability. The United Nations Human Rights Council has proposed several principles for supporting health damage from hazardous chemicals [27]. According to this, the state is primarily responsible for protecting the right to immediate and effective relief. Courts of FRA and JPN similarly stated that the state was liable [8,11]. As for who will share the relevant budget, it is correct for the state to take practical responsibility. It is also true that the entire industry should bear responsibility for the compensation due to the undisputed fact that they have reaped the benefits from the use of asbestos. Additionally, the imposition of additional collections on asbestos-related businesses is considered to be the most consistent with the principles of justice, the legal principles of the obligation to compensate for damage, and the polluter-pays-principle. Social justice and fairness also put the responsibility for the compensation of damages caused on the person who creates the harm [8].

### 4.2. Qualification

NLD requires that victims be alive and to have lived at least 10 consecutive years in NLD, 10 to 60 years prior to the application. This can be evaluated as an unreasonable qualification, considering that most of the causes of mesothelioma are related to asbestos and the fact that mesothelioma can develop even with a very small amount of exposure to asbestos if there is a sufficient latent period of 20 to 40 years [23,28]. NLD is also the only country where the victim cannot reapply for ARD compensation other than for the previously recognized ARD. The occurrence of a new disease is a fresh instance of damage and pain. Nevertheless, the fact that reapplication is not allowed may be considered as a reflection of a passive attitude towards the protection of the victims’ rights as compared to other countries. 

An application has to be made based on certain criteria and within a time limit to qualify. The intended beneficiary loses his rights if he does not exercise the same within a specified time limit. In other words, an extinctive prescription or a period for extinguishing the right itself exists. The extinctive prescription seeks to stabilize the social order and prevent administrative inefficiencies resulting from difficulties in proving past facts. It is to prevent the waste of social resources as an inducement to prevent the neglect of exercise of rights. However, there is a fundamental question of whether depriving the rights of the right holder serves justice [29]. Therefore, it is important to keep these two conflicting goals in balance. This may differ from country to country for several reasons, such as whether the appropriate government intervention is being implemented, the search for victims until compensation is made, and how well the system is being promoted. Depending on the country’s situation, further study is needed to determine whether the limited period set for the somewhat conflicting objectives of protecting rights holders and preventing unnecessary waste of social resources is reasonably satisfied.

### 4.3. Expiration Date

The validity period differs from country to country due to differences in the type of benefit (pension or lump sum) and purposes by the system. First, NLD and GBR, which provide lump-sum payments, are not subject to the issue of the validity period. In the other countries where the form of benefits consists of treatment expenses, pensions, etc., there is still the question of setting the length of the compensation period. Under the goal of complete compensation, FRA does not set the expiration date so as to compensate for all damages that occur until death as well as for the period from the incidence of damage to the end of treatment. BEL provides a mixture of lump sum and pension types, but since the pension is a means to support the lives of victims with permanent disabilities that occurred after treatment was terminated, the expiration date was not set. JPN and KOR limit the duration because they aim to provide assistance only for the duration of treatment of the disease. JPN and KOR uniformly set five years with the goal of subsidizing medical expenses and living expenses only during the period of treatment, and do not offer support after the treatment is completed. This shows that JPN and KOR have a passive attitude in terms of damage recovery, compared to FRA and BEL who set the validity period as lifetime maintenance. JPN and KOR can extend the validity period of the system further if the necessity to continue treatment is proved through a renewal application. 

### 4.4. Disease

A comprehensive comparative analysis of recognized diseases revealed that all countries were slow in updating the list of recognized diseases based on the carcinogenicity evaluation of the International Agency for Research on Cancer (IARC). According to IARC, there is sufficient evidence to implicate asbestos as one of the causative agents for the development of mesothelioma, lung cancer, laryngeal cancer, and ovarian cancer in humans. Additionally, there is limited evidence suggesting the role of asbestos in causing pharyngeal cancer, stomach cancer, and colorectal cancer in humans [30]. As is well known, asbestosis and diffuse pleural thickening are representative diseases caused by exposure to asbestos [31,32,33]. It is considered reasonable to include mesothelioma, lung cancer, laryngeal cancer, and ovarian cancer as asbestos has been evaluated by IARC as an agent with sufficient evidence of causing these diseases [12]. As with various occupational compensation systems and FRA’s asbestos victim relief scheme, it is necessary to operate the compensation system as a mixed type which means classifying recognized diseases in a list and evaluating other diseases individually to determine whether the applicant’s disease is related to asbestos [34].

### 4.5. Type of Benefits

In JPN and KOR benefits cannot be received after treatment is terminated. Such a structure burdens the victim with the lifelong pain caused by disability although no further treatment can be given. Besides, structurally, it is designed to induce victims to extend treatment because there is no compensation that they can receive after treatment is over [35]. Therefore, if there is an expiration date like JPN and KOR, it seems appropriate to provide benefits that the victim can continue to receive even after the treatment is terminated.

In the asbestos victim relief schemes of all countries, the government and compulsory insurance pay first for treatment. Subsequently, FRA pays all the remaining expenses while only part payment is made by JPN, BEL, and KOR. According to Organization for Economic Co-operation and Development (OECD) statistics, the coverage of government and compulsory insurance varies by country: FRA 83% (2017), JPN 84% (2017), BEL 76% (2017), NLD 82% (2017), GBR 79% (2017), and KOR 59% (2017) [36]. Household out-of-pocket payments as a proportion of the current expenditure on health are as follows: FRA 9.2% (2018), JPN 12.7% (2017), BEL 19.1% (2018), NLD 10.8% (2018), U.K 16.7% (2018), KOR 32.5% (2018) [36]. JPN, BEL, and KOR are paying part of the treatment cost, but it was not possible to know the exact effect of this on the proportion of household out-of-pocket payments due to data limitations. However, this ratio for KOR could be estimated to decrease from 32.5% (2018) to 14.5%–17.1% (2017) [37]. In JPN and BEL too, this ratio can be predicted to decrease at a rate similar to KOR because the method of supporting treatment costs is the same as KOR. In summary, the treatment cost burden from the victim’s perspective could be estimated in the descending order of FRA, JPN, BEL≈NLD, and GBR≈KOR. Therefore, it seems that NLD and GBR which do not support treatment expenses at all should reduce the burden on the victim by providing benefits related to treatment expenses. As KOR currently lacks the level of government and compulsory insurance, it is thought that it is necessary to expand the support for medical expenses.

### 4.6. Judgement Criteria

Making decisions based solely on medical criteria can be problematic. Since malignant mesothelioma is mostly caused by asbestos, there is no controversy over its recognition solely by diagnosis. However, similar decisions involving lung cancer or asbestosis can be a problem because there are many grey areas if based on medical evaluation alone. Since chrysotile has a low bio-persistency, very little remains in the tissues over time. Additionally, only 36% of the lung cancers in the group exposed to asbestos are accompanied by asbestos lungs [38]. These facts lead to the presumption that there are many cases where recognition is not obtained even though the disease is strongly related to asbestos. Therefore, KOR should improve the current system by combining evaluation with exposure criteria. 

Countries that have implemented exposure criteria as tools for evaluation are currently conducting only occupational exposure-based assessments. The implementation of non-occupational exposure assessments has not been widespread. However, non-occupational exposure to asbestos cannot be ignored [39,40]. Therefore, studies on how to evaluate non-occupational asbestos exposure are necessary. Additionally, there has recently been a controversy over the suitability of the Helsinki criteria for the evaluation of lung cancer caused by asbestos [41,42,43,44]. 

A long-standing controversy regarding the criteria is the proof of causality. The causal relationship judgment for environmental damage is characterized by the difficulty faced by a victim in proving this relationship due to the limitations of the current level of scientific and medical knowledge and the ubiquity of information. In the case of the asbestos victim relief schemes, demanding a strict degree of scientific verification the current lack of scientific understanding and evidence is contrary to the basic idea of fairness and justice. The absence of damage evidence should not be regarded as evidence of the absence of damage, and it should be approached from a practical point of view [45,46].

### 4.7. Appeals

Ensuring an opportunity for appeals in the case of disapproval of an application is ultimately necessary in terms of the goal for a just outcome. The decision could be unjust due to lack of evidence, lack of verification efforts, and subjective factors influencing the judges. In this regard, FRA, BEL, and GBR, who do not have a system related to appeals, can be assessed to have systems that are unfair in terms of securing the rights of victims. However, there was also a limit to grasping whether the system was guaranteed to ensure fair results simply by the number of opportunities available. If a single decision process provides sufficient opportunity for the exchange of arguments and deliberation by several experts, it is more equitable than simply guaranteeing the availability of multiple appeals to the applicant. Additionally, the degree of ease of filing an objection through a court administered litigation may vary from country to country in terms of the cost and the burden of proof from the perspective of the victims. Therefore, beyond the number of opportunities for appeals, it seems necessary to further analyze the number of proceedings in administrative litigation, to compare the results of administrative litigation and the asbestos victim relief schemes, and to scrutinize the ease of administrative litigation country by country.

### 4.8. Recognition Rate

The reason why GBR’s recognition rate is over 100% is that the year of application and the year of recognition are different. GBR’s total recognition rate is high at 102% because only mesothelioma is a recognized disease in the asbestos victim relief scheme. Only diagnosis is required without any medical and exposure criteria. KOR is unique in that the recognition rate for asbestosis and diffuse pleural thickening is different from that of other countries. Asbestosis has a relatively high recognition rate. This is a result of dividing it into three grades thus lowering the hurdles in the judgment criteria, and providing differential compensation amounts. On the contrary, the recognition rate for diffuse pleural thickening is relatively low due to the stringent demand for medical criteria.

### 4.9. Number of Recognized Claims

Analysis of the total number of recognized claims by combining the conventional occupational compensation scheme and the asbestos victim relief scheme revealed that NLD has a relatively lower number (between 7 times up to 20 times lower) compared to other countries. Since this tendency is the same for all diseases, NLD may be termed passive in its compensation of the ARD victims.

Compared to other countries, KOR’s ratio of recognized claims between the conventional occupational compensation scheme and the asbestos victim relief scheme was large, regardless of the type of disease. This is related to KOR’s compressed economic growth with a history of the social security system also developing at a slow pace [47]. In other words, despite occupational asbestos exposure, many workers are not eligible for the worker’s compensation system due to the history of low coverage of the system. This can be a problem because there are differences in the status of the victim depending on the system to which the victim belongs, the dignity of the victim, the scope and amount of compensation, and the entity responsible for the fund. KOR can refer to GBR. GBR operates the Diffuse Mesothelioma Payment Scheme (DMPS) for those who were exposed to occupational asbestos but are unable to trace the employers or the insurers due to various limitations. The difference between DMPS and the ‘2008’ scheme, which is an asbestos victim relief scheme, is that the ’2008’ scheme does not require proof of negligence, but DMPS requires detailed evidence including witness statements, date of diagnosis, medical records, full employment history, and evidence of failed attempts to track previous employers or underwriters. Due to these requirements, in the case of DMPS, the compensation amount is much higher than that of the ‘2008 scheme’, but there is also a likelihood that the application may be rejected [48].

### 4.10. Compensation Amount

The question of how much compensation is adequate always arises. FRA, which aims for complete compensation, is free from such conflicts. There is also no difference in compensation even if the route of exposure is different. At the time of the establishment of FIVA, to underline its legitimacy of purpose, the French Minister for Employment and Solidarity stated that it was imperative for the community to pay equitable compensation to victims in the name of national solidarity [11,49]. This means that it is justified to compensate them because the tragedy of asbestos victims is not simply caused by a specific number of perpetrators but is the result of a tremendous contribution to national development along with industrial development, with the entire community enjoying all the benefits. It is well known that asbestos has been used in various ways across industries in most countries. The whole society as an economic community has directly or indirectly obtained economic benefits from the use of asbestos; the state has not taken appropriate measures to prevent damages even though they have recognized or could have recognized the risk of damage caused by asbestos. Considering this, the FRA’s dispute resolution approach based on national solidarity and state responsibility can serve as a reference point for many countries. [11,50].

### 4.11. Limitations of Research and Necessity of Follow-Up Research

This paper considers several social systems and factors for the comparative analysis of the asbestos victim relief schemes. Nevertheless, it is unfortunate that some countries’ analysis was only partially provided due to the limitations of data disclosure and scope (especially, FRA’s judgment criterion and disease-specific number of recognized claims, BEL’s number of occupational ARD recognized claims, and average compensation amount per person). Additionally, even with the same asbestos victim relief scheme, the method of operating the system differs from country to country. For example, one country covers occupational compensation and asbestos victim relief. Another simply operates them by statistically classifying exposure. Other countries have very strict standards for their conventional occupational compensation scheme, which leads to many applicants opting for the asbestos victim relief scheme. Therefore, it was impossible to compare and analyze the number of recognized claims for only asbestos victim relief. For this reason, a comparison of recognized claims has been attempted by integrating asbestos victim relief with the occupational ARD compensation system. It is a limitation that the ratio of all ARD dispute resolution methods in a country has not been analyzed by litigation, occupational ARD compensation system, asbestos victim relief, and unresolved cases. Another limitation is that the average compensation amount per person has not been more clearly defined in consideration of the medical payment system and the welfare system for the disabled.

The system does not stand still. In the triangle of various stakeholders namely victims, funders, and the government, it continues to change through a tug of war. It is expected that the number of victims of ARD will continue to increase over a long period, and their demands for compensation will also expand. However, it cannot be properly resolved through the conventional legal system due to the characteristics of ARD. Currently, only six countries are implementing asbestos victim relief schemes, and the actual period for which these systems have been in existence is short. In that respect, it is thought that a lot of research related to these systems is needed in the future before it is too late. It is expected that more meaning can be gained by the analysis of recognized claims through the analysis of the coverage of all ARD dispute resolution methods by litigation, conventional occupational compensation scheme, asbestos victim relief, and unresolved claims. As mentioned above, if the average compensation amount per person including the medical payment system and the welfare system for the disabled can be calculated, a more objective comparison will be possible. Additionally, it is anticipated that research would be conducted on methods to tackle the recent controversy over the Helsinki criteria and the fact that non-occupational exposure cannot be measured by these exposure criteria. This can be helpful to those who operate related policies or related asbestos victim relief schemes in the future. In particular, the relevant stakeholders will be able to improve their understanding of the system, and better system development can be expected through an exchange of opinions on topics and principles that need discussion.

## 5. Conclusions

A comprehensive comparison and analysis of the asbestos victim relief schemes of six countries gives us the universality and specificity of these systems and highlights the factors that influenced them. In terms of the number of recognized claims and the compensation amount, which is the outcome of the system, FRA is the most active. The differences between countries were largely caused by the purpose of the institutional design when examining the relationship with the right to litigation (complete compensation (FRA, BEL) or burden reduction (JPN, NLD, GBR, KOR)). The differences were also influenced by the level of qualification, the existence of an expiration date, type of disease, type of benefit, level of judgment criteria, the existence of appeals, and recognition rate. Furthermore, the findings of this analysis were able to suggest directions and offer advice. Concerning the diseases, the introduction of open type systems and the expansion of disease types are necessary. In the coverage on types of benefits, it is felt that victims should be permitted to continue to receive benefits after treatment ends and that it would be better if treatment expenses are guaranteed. Finally, an overall review of judgment criteria is necessary, along with the need for exposure assessment, resolution of the controversy over the Helsinki criteria, and addressing the absence of non-occupational exposure assessment.

## Figures and Tables

**Table 1:** Comparison of the asbestos victim relief schemes across countries.

		FRA	JPN	BEL	NLD	GBR	KOR
**Name**		Fund for Compensation of Asbestos Victims (FIVA)	Asbestos Health Damage Relief System	Asbestos Fund (AFA)	Non-work-related Compensation for Asbestos Victims (TNS)	Diffuse Mesothelioma Payments (the ‘2008’ scheme)	Asbestos Injury Relief System
**The office concerned**		Ministry of Solidarity and Health	Ministry of Environment	Ministry of Social Affairs and Public Health	Ministry of Infrastructure and Water Management	Department for Work and Pensions	Ministry of Environment
**When established**		2002	2006	2007	2007	2008	2011
**Occupational ARD compensation system**		Worker’s Compensation System + FIVA	Worker’s Compensation System	Worker’s Compensation System	Compensation for Asbestos Victims (TAS)	Worker’s compensation system, Diffuse Mesothelioma Payment Scheme (DMPS)	Worker’s Compensation System
**Litigation rights after compensation**		impossible	possible	impossible	possible	possible	possible
**Budget**		worker’s compensation system + government	new fund (worker’s compensation subject + asbestos-related businesses + government)	worker’s compensation system + government + social security system + other donations	social insurance bank (SVB)	recovery of benefits	new fund (worker’s compensation subject + asbestos-related businesses + government)
**Qualification**	**When alive**	up to 10 years (from the date of the first medical certificate relevant asbestos)	not required	not required	at least 10 consecutive years in NLD, 10 to 60 years prior to the application	up to 1 year (from the date of diagnosis)	not required
	**After death**		up to 15 years (from date of death)	up to 6 months (from date of death)	impossible	up to 1 year (from date of death)	up to 15 years (from date of death)
	**Reapply after recognized**	possible	possible	possible	impossible	only mesothelioma	possible
	**Apply after compensated by occupational ARD compensation system**	possible (if received compensation from worker’s compensation system not from FIVA).	impossible	possible	impossible	impossible	impossible
**Expiration date**		no limit	5 years	no limit	lump sum	lump sum	5 years
**Disease**	**Open type**	○	✕	✕	✕	✕	✕
	**List**	mesothelioma, lung cancer, asbestosis, diffuse pleural thickening, pleural plaque, pericardial plaque, exudative pleuritis, pleural tumors	mesothelioma, lung cancer, asbestosis, diffuse pleural thickening	mesothelioma, lung cancer, laryngeal cancer, asbestosis, diffuse pleural thickening	mesothelioma, asbestosis	mesothelioma	mesothelioma, lung cancer, asbestosis, diffuse pleural thickening

**Table 2:** Type of benefit.

	FRA	JPN	BEL	NLD	GBR	KOR
Recognized when alive	(i) loss of income (ii) medical expenses (total) (iii) other expenses (ex. house repair) (iv) disability compensation (v) psychological compensation (vi) physical compensation (ex. pain) (vii) loss of pleasure (ex. inability to do leisure activities) (viii) aesthetic damage (ex. scar)	(i) medical expenses (partial) (ii) subsistence allowance (iii) funeral expenses	(i) medical expenses (partial) (ii) lump sum (only mesothelioma) (iii) subsistence allowance (flat rate for mesothelioma, in proportion to % of the disability for the other disease) (iv) attendance allowance	lump sum	lump sum	(i) medical expenses (partial) (ii) subsistence allowance (iii) funeral expenses
Recognized after death	(i) loss of income (ii) medical expenses (total) (iii) other expanses (iv) lump sum (v) funeral expenses	(i) lump sum (ii) funeral expenses	(i) lump sum (ii) funeral expenses			(i) lump sum (ii) funeral expenses

**Table 3:** Medical criteria.

	Mesothelioma	Lung cancer	Asbestosis	Diffuse Pleural Thickening
FRA	Check only the diagnosis	Nondisclosure		
JPN	Check only the diagnosis	One of 1–3 is satisfied. 1. Pleural plaque (on chest X-ray or CT *) + fibrosis (on chest X-ray) 2. One of the following satisfied (1) pleural plaque (on chest X-ray and CT *) (2) on chest CT *, enlargement of pleural plaque is more than 1/4 of the inner chest wall on either side. 3. One of the following satisfied (1) 5000 or more asbestos bodies/1 g (dry tissue) (2) 20,000 or more asbestos fibers (5 μm or more)/1 g (dry tissue) (3) 50,000 or more asbestos fibers (1 μm or more)/1 g (dry tissue) (4) 5 or more asbestos bodies/1 mL (bronchoalveolar lavage fluid) (5) asbestos body on lung tissue section	Both 1–2 are satisfied. 1. Fibrosis (on chest X-ray) 2. One of the following satisfied (1) VC ** less than 60% (2) VC ** 60–80%, FEV1 *** / FVC **** less than 70%, FEV1 *** less than 50% (3) VC ** 60–80% + PaO2 ***** less than 60 torr or significant increase of AaDO2 ********	One of 1–3 satisfied 1. On chest X-ray, if one side thick, more than 1/2 of the inner chest wall + On chest CT *, not a pleural plaque 2. On chest x-ray, if both side thick, more than 1/4 of the inner chest wall + On chest CT *, not a pleural plaque 3. If 1, 2 not known by pleural effusion, (1), (2) and one of (3), (4), (5) satisfied (1) pleural effusion irregularity (2) crow’s feet sign or round atelectasis (3) air in pleural effusion (4) stabilization of pleural effusion volume (5) decrease in thoracic capacity
BEL	Check only the diagnosis	Electron microscope: 2 million or more asbestos fibers (5 μm or more)/1 g (dry tissue) Optical microscope: 5 or more asbestos bodies/1 mL (bronchoalveolar lavage fluid), 5000 or more asbestos bodies/1 g (dry tissue)		
NLD	Check only the diagnosis		Both 1–2 are satisfied. 1. On chest CT *, fibrosis more than 5% 2. One of the following satisfied (1) FVC **** less than 70% (2) DLCO ****** less than 70% (3) VO2max ******* less than 22 mL/kg/min	
GBR	Check only the diagnosis			
KOR	Check only the diagnosis	One of 1–3 is satisfied. 1. Asbestosis 2. Pleural plaque 3. One of the following satisfied (1) 5000 or more asbestos bodies/1 g (dry tissue) (2) 20,000 or more asbestos fibers (5 μm or more)/1 g (dry tissue) (3) 50,000 or more asbestos fibers (1 μm or more)/1 g (dry tissue) (4) 5 or more asbestos bodies/1 mL (bronchoalveolar lavage fluid)	Fibrosis (on chest CT *)	One of 1–2 is satisfied. 1. All of the following satisfied (1) on chest CT *, the length of the chest wall is more than 5 cm (2) on chest CT *, the length in the craniocaudal direction is 8 cm or more (3) on chest CT *, more than 3 mm thick 2. One of the following satisfied (1) FVC **** less than 50% (2) FEV1 *** less than 45% (3) DLCO ****** less than 45%

* Computed tomography. ** vital capacity. *** forced expiratory volume in 1 second. **** forced vital capacity. ***** partial pressure of arterial oxygen. ****** diffusion capacity of carbon monoxide. ******* maximal oxygen uptake. ******** alveolar-arterial oxygen difference.

**Table 4:** Medical criteria for recognition of asbestosis in South Korea.

Radiological Criteria				
Early type		One of 1–8 pulmonary fibrosis findings is satisfied + less than 1/3 of the outside of the lung filled.	1. Subpleural dotlike or branching opacities 2. Subpleural curvilinear opacities 3. Parenchymal bands 4. Intralobular interstitial thickening 5. Interlobular septal thickening 6. Bronchiectasis 7. Ground-glass opacities. 8. Honeycombing	
Progressive type		One of 1–8 pulmonary fibrosis findings is satisfied + more than 1/3 of the outside of the lung filled.		
**Pulmonary Function Test Criteria**				
**Grade**		FVC *	FEV1 **	DLCO ***
Normal	One of FVC *, FEV1 **, DLCO *** is satisfied	More than 80%	More than 80%	More than 75%
Mild		50%–80%	45%–80%	45%–75%
Severe		Less than 50%	Less than 45%	Less than 45%
**Final criteria (radiological criteria + pulmonary function test criteria)**				
Grade 1		Radiological criteria: early type + PFT **** criteria: normal		
Grade 2		One of 1-2 is satisfied	1. Radiological criteria: early type + PFT **** criteria: mild 2. Radiological criteria: progressive type + PFT **** criteria: normal	
Grade 3		One of 1–3 is satisfied	1. Radiological criteria: early type + PFT **** criteria: severe 2. Radiological criteria: progressive type + PFT **** criteria: mild 3. Radiological criteria: progressive type + PFT **** criteria: severe	

* Forced vital capacity. ** forced expiratory volume in 1 second. *** diffusion capacity of carbon monoxide. **** pulmonary function test.

**Table 5:** (a) Comparison of the number of recognized claims of asbestos victim relief (all periods), (b) Comparison of the total number of recognized claims of conventional occupational compensation scheme and asbestos victim relief scheme (2016–2018).

	Mesothelioma		Lung Cancer		Asbestosis		Diffuse Pleural Thickening		Total	
	Actual	Corrected	Actual	Corrected	Actual	Corrected	Actual	Corrected	Actual	Corrected
(a)										
FRA *	-	-	-	-	-	-	-	-	85,889	2334
**JPN**	11,860	434	1911	70	78	4	163	9	14,012	518
**BEL**	2287	53			309	7	513	12	3109	72
**NLD** **	1213	22			1	0			1214	22
**GBR**	5220	73							5220	73
**KOR**	1074	398	623	231	**Total**		2	1	**Total**	
					2436	902			4137	1553
					**Excluding asbestosis grades 2 and 3**				**Excluding asbestosis grades 2 and 3**	
					243	90			1944	721
**(b)**										
FRA ***	-	-	-	-	-	-	-	-	10,843	1571
**JPN**	4129	655	1541	245	206	33	212	34	6088	966
**NLD**	1431	88			108	7			1539	95
**GBR**	7900	411	630	33	2960	154	1380	72	12,870	670
**KOR**	276	307	326	362	**Total**		2	2	**Total**	
					1076	1196			1680	1867
					**Excluding asbestosis grades 2 and 3**				**Excluding asbestosis grades 2 and 3**	
					158	176			762	847

* The number of newly recognized cases (85,889) was estimated by multiplying the number of newly applied cases (2002–2018, 102,249) by the recognition rate of all cases (84%). ** The years (2016 and 2017) were excluded. *** Only FIVA was calculated because the recognized claims of worker’s compensation system are likely to be calculated twice in FIVA. The number of newly recognized cases (10,843) was estimated by multiplying the number of newly applied cases (2016–2018, 11,915) by the recognition rate of all cases (91%).

**Table 6:** Comparison of the ratio of recognized claims between conventional occupational compensation scheme and asbestos victim relief (2016–2018).

	Mesothelioma			Lung Cancer			Asbestosis			Diffuse Pleural Thickening			Total		
	COC *	AVR **	COC */AVR **	COC *	AVR **	COC */AVR **	COC *	AVR **	COC * /AVR **	COC *	AVR **	COC * /AVR **	COC *	AVR **	COC * /AVR **
**JPN**	1638	2491	0.66	1098	443	2.48	188	18	10.44	137	75	1.83	3061	3027	1.01
NLD ***	353	147	2.40				38	1	38				391	148	2.64
**GBR**	6640	1260	5.27										6640	1260	5.27
**KOR**	39	237	0.16	37	289	0.13	57	**Total**		0	2	0	133	**Total**	
								1019	0.06					1547	0.09
								**Excluding asbestosis grades 2 and 3**						**Excluding asbestosis grades 2 and 3**	
								101	0.56					629	0.21

* Conventional occupational compensation scheme; ** asbestos victim relief; *** the years (2016 and 2017) were excluded.

**Table 7:** Comparison of the average compensation amount per person from the asbestos victim relief schemes divided by the median income (all periods).

	Mesothelioma	Lung Cancer	Asbestosis	Diffuse Pleural Thickening
**FRA**	4.60	4.92	1.75	1.03
**JPN**	1.35			
NLD *	0.73	0.78		
**GBR**	1.03			
**KOR**	1.46	1.45	**Total**	1.06
			0.40	
			**Grade 1**	
			1.28	
			**Grade 2**	
			0.48	
			**Grade 3**	
			0.20	

* The years (2016 and 2017) were excluded.

## Data Availability

Not applicable.
